# Ketogenic diet in action: Metabolic profiling of pyruvate dehydrogenase deficiency

**DOI:** 10.1016/j.ymgmr.2023.100968

**Published:** 2023-03-20

**Authors:** Eri Ogawa, Takako Hishiki, Noriyo Hayakawa, Hisato Suzuki, Kenjiro Kosaki, Makoto Suematsu, Toshiki Takenouchi

**Affiliations:** a Department of Pediatrics, Keio University School of Medicine, Tokyo, Japan; b Department of Biochemistry, Keio University School of Medicine, Tokyo, Japan; c Clinical and Translational Research Center, Keio University School of Medicine, Tokyo, Japan; d Center for Medical Genetics, Keio University School of Medicine, Tokyo, Japan; e Central Institute for Experimental Animals, Kawasaki, Kanagawa, Japan

**Keywords:** Metabolome, Whole genome sequencing, Pyruvate dehydrogenase deficiency, Ketogenic diet

## Abstract

The pyruvate dehydrogenase complex serves as the main connection between cytosolic glycolysis and the tricarboxylic acid cycle within mitochondria. An infant with pyruvate dehydrogenase complex deficiency was treated with vitamin B1 supplementation and a ketogenic diet. These dietary modifications resolved the renal tubular reabsorption, central apnea, and transfusion-dependent anemia. A concurrent metabolome analysis demonstrated the resolution of the amino aciduria and an increased total amount of substrates in the tricarboxylic acid cycle, reflecting the improved mitochondrial energetics. Glutamate was first detected in the cerebrospinal fluid, accompanied by a clinical improvement, after the ketogenic ratio was increased to 3:1; thus, glutamate levels in cerebrospinal fluid may represent a biomarker for neuronal recovery. Metabolomic analyses of body fluids are useful for monitoring therapeutic effects in infants with inborn errors of carbohydrate metabolism.

## 1 Introduction

Pyruvate dehydrogenase E1-alpha deficiency (PDHAD, OMIM #312170) is a prototypic treatable inborn error of central carbohydrate metabolism due to defective pyruvate dehydrogenase E1-alpha, which is encoded by *PDHA1* on Xp22.12 [1]. Pyruvate dehydrogenase complex functions as the main connection between cytosolic glycolysis and the tricarboxylic acid (TCA) cycle within mitochondria. It follows that PDHAD patients cannot utilize pyruvate as an energy source, and the defective energy production leads to various systemic manifestations. The central nervous system phenotype for PDHAD includes developmental delay, intellectual disability, myotonic abnormality (hypotonia and hypertonia), epilepsy, dystonia, ataxia, ventriculomegaly, and corpus callosum abnormalities [2,3]. The mortality rate is as high as 40% in boys, which is higher than that for girls, while the long-term intellectual developmental prognosis is mostly moderate to profound and poorer in girls than in boys [2].

The mainstay of treatment in PDHAD is carbohydrate intake restriction and a high fat diet, a so-called “ketogenic diet.” The purpose of the ketogenic diet is to provide ketone bodies as alternative fuel to the TCA cycle via β-oxidation. An improvement in lactic acidosis as a clinical benefit of a ketogenic diet was first described more than four decades ago [4]. Increasingly, a ketogenic diet is being recognized as an effective therapy for epilepsy, delayed psychomotor development, sleep disturbance, and so on [2,3,[5], [6], [7], [8], [9]]. However, it has remained unknown how the ketogenic diet affects metabolism within the TCA cycle, which is the source of cellular energy production, and energetics in multiple organ systems.

## 2 Case report

The proposita was born at 38 weeks of gestation by scheduled cesarean section because of a history of prior cesarean delivery. The pregnancy was uncomplicated until 28 weeks of gestation. At 33 weeks of gestation, intrauterine growth retardation and ventriculomegaly were noted. The patient's parents were non-consanguineous, and there was no family history of inherited metabolic disorders. Birth weight was 2.492 g (−1.0 SD), head circumference 31.5 cm (−1.1 SD), and body length 43.0 cm (−1.0 SD). The Apgar scores were 4 and 8 at 1 min. and 5 min., respectively. The patient exhibited severe hypotonia and required mechanical ventilation for 4 days, and she remained in the neonatal intensive care unit for 33 days.

The laboratory test results are summarized in the Table 1. They showed markedly elevated blood and cerebrospinal fluid (CSF) pyruvate and lactate levels. Serum biochemistry data revealed persistent hyperlactatemia and hyperpyruvatemia, and elevated ammonia and alanine levels. Brain magnetic resonance imaging revealed loss of white matter and agenesis of the corpus callosum. The patient had central apnea and spasticity of the extremities. The overall clinical course and laboratory data triggered suspicion of PDHAD.

**Table 1:** Chronological changes in biochemical trends in relation to supplement and nutritional intake of ketogenic diet.

	**Days of treatment**	0	1	3	6	8	10	14	21	36	52
**Supplement**											
	Vitamin B1 [mg/day]	–	100	300	450	100	100	100	100	100	100
**Nutritional Intakes of ketogenic diet**											
	Proteins [grams/kg]	–	–	–	–	2.41	2.41	2.41	2.36	2.36	2.34
	Lipids [grams/kg]	–	–	–	–	8.74	8.74	8.74	10.34	10.34	11.2
	Carbohydrates [grams/kg]	–	–	–	–	6.73	6.73	6.73	3.15	3.15	1.37
	Calculated ketogenic ratio	–	–	–	–	0.96:1	0.96:1	0.96:1	1.9:1	1.9:1	3.0:1
**Biochemical Trends**											
Cerebrospinal fluid	Lactate (reference:< 16 mg/dL)	72.1							32.3		19.9
	Pyruvate (0.63–0.77 mg/dL)	8.81							4.22		2.30
Serum	Lactate (3.7–16.3 mg/dL)	35.8				34.1		15.8	14.4		16
	Pyruvate (0.30–0.90 mg/dL)	2.92				3.16		1.26	0.89		0.85
	Alanine (253.6–601.9 nmol/L)	615.9				732.0		312.8	441.2		350.8
	Hydroxy proline (< 19.7 nmol/L)	54.4				45.1		34.9	35		30.8
	Ammonia (< 60 μmol/L)	103		32	46		34		24		18
	Ketone bodies (26–122 μmol/L)	93						272	1120		1889
	β-hydroxybutyrate (0–76 μmol/L)	54						162	773		1325
Whole blood	Hemoglobin (10.5 ± 1.2 g/dL)	8.7		7.3	7.1		10.3	9.7	9.9	10.7	9.7
	Reticulocyte (0.5–2.5%)	3.6		3.6	3.6	⁎				1.7	

⁎ Packed red blood cell was transfused on the 8th day of treatment with vitamin B1 supplementation.

Since a subgroup of PDHAD patients have been reported to respond to vitamin B1 supplementation [10,11], the patient was empirically started on high-dose vitamin B1 at 450 mg/day, and it resulted in improvement of the hyperammonemia. However, progressive anemia with a hemoglobin concentration of 8.7 g/dL, then at 7.1 g/dL, persisted, and she was treated with packed red blood cell transfusions on the 8th day of treatment with the vitamin B1 supplementation. After genetic diagnosis confirmed the suspicion of PDHAD, the patient was started on a ketogenic diet with a ketogenic ratio (defined as the ratio by weight of lipid content to carbohydrate plus protein content in the diet) of 1:1. After increasing the ketogenic ratio to 3:1, the patient exhibited signs of clinical and biochemical improvement manifested by resolution of the central apnea and transfusion-dependent anemia, resolution of the lactic acidemia in both serum and CSF, and improvement of the hyperalaninemia.

The patient's psychomotor development was delayed. At 6 months of age, she fixated and followed objects and cooed. She required multiple antiepileptic medications for a symptomatic West syndrome. At 18 months of age, the patient was able to continue the ketogenic diet without adverse effects. She acquired head control and visual pursuit but was unable to sit without support.

## 3 Molecular analysis

### 3.1 Genetic analysis

Our research protocol was approved by the ethics committee of Keio University School of Medicine, Tokyo, Japan (approval number: 20110262). Written consent to performing a genetic analysis was obtained from the parents. Peripheral blood samples were obtained from the patient and her biological parents. DNA was extracted by the standard phenol extraction method. A whole genome sequencing in trio identified a de novo heterozygous missense mutation in *PDHA1*: chrX(GRCh37):g.19369472G > T, NM_001173454.1:c.479G > T, p.(Gly160Val). Sanger sequencing confirmed the results (Fig. 2). This mutation was absent in 3552 healthy Japanese individuals [12] and had never been reported in the Genome Aggregation Database (gnomAD, http://gnomad.broadinstitute.org/). The result of an in silico analysis using PolyPhen-2 was “deleterious,” and highly conserved among many non-human species [13]. The PROVEAN (Protein Variation Effect Analyzer) score was −8.121 [14]. According to the updated criteria for the pathogenicity of the variant by the American College of Medical Genetics [15], the mutation was considered “pathogenic.” A transcriptome analysis of the patient's RNA extracted from a peripheral blood sample showed expression of wild-type alleles and complete absence of expression of the mutant alleles (data not shown). Based on the assumption that X-inactivation was non-skewed, the mutant allele was inactivated in 50% of the peripheral lymphocytes, and it was active in the other 50%. The mutant transcript was not detected, probably because it was unstable and was therefore degraded by nonsense-mediated mRNA decay.

### 3.2 Metabolomic analysis

To assess the impact of the ketogenic diet on amino acid and central carbon metabolic pathways, urine, CSF, and arterial whole blood samples were collected when the diagnosis was made, after the start of vitamin B1 supplementation, 7 days after starting the 2:1 ketogenic diet, and 10 days after switching to the 3:1 ketogenic diet. The serum was separated by centrifugation after the blood clotted. To avoid metabolite degradation, all samples were immediately frozen at −80 °C until analyzed. Quantitative capillary electrophoresis-electrospray ionization mass spectrometry (CE/ESI/MS, Agilent Technologies, Santa Clara, CA) was used to measure the metabolites as previously described [[16], [17], [18]].

**Fig. 1:**
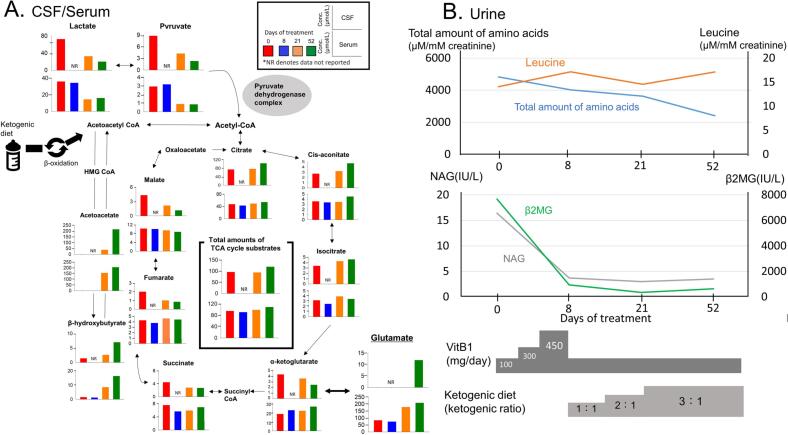
Serial metabolome data of the TCA cycle in CSF and serum and amino acids in urine samples. A: The total amount of TCA cycle substrates represents the sum of the individual concentrations. Note that the total amounts of TCA cycle substrates and glutamate increased both in the CSF and serum in proportion to the ketogenic ratio of the ketogenic diet. B: The top panel shows chronological changes in the total urinary amounts of all measured amino acids, adjusted by the concurrent creatinine concentration. The middle panel shows chronological changes in the relative urinary excretion of β-2-microglobulin (β2MG) and *N*-acetyl-β-D-glucosaminidase (NAG) after the start of vitamin B1 supplementation and the ketogenic diet. The horizontal axes represent days of treatment, with details shown in the bottom panel. Note the steep decline in urinary excretion of β-2-microglobulin after the start of vitamin B1 supplementation, reflecting improvement of proximal tubular function.

The serial metabolomic serum, CSF, and urine analyses at the multiple time points during diagnosis and after the start of vitamin B1 supplementation and ketogenic diet confirmed the diagnosis of PDHAD. The ketogenic diet induced robust metabolic changes outside the TCA cycle but not within it (Fig. 1A). First, both serum and CSF lactate and pyruvate levels were lower, reflecting the decreased glucose and protein intake. Second, the increases in the ketone bodies acetoacetate and β-hydroxybutyrate likely reflected increased fatty acid intake. Although there were no overt changes in any of the metabolites within the TCA cycle, there was an increase in the total amounts of TCA cycle substrates that was in proportion to the ketogenic ratio. Third, there was an increase in serum glutamate in proportion to the ketogenic ratio. Glutamate was first detected in CSF 31 days after the increase in ketogenic ratio from 2:1 to 3:1, i.e., 52 days after the start of treatment.

**Fig. 2:**
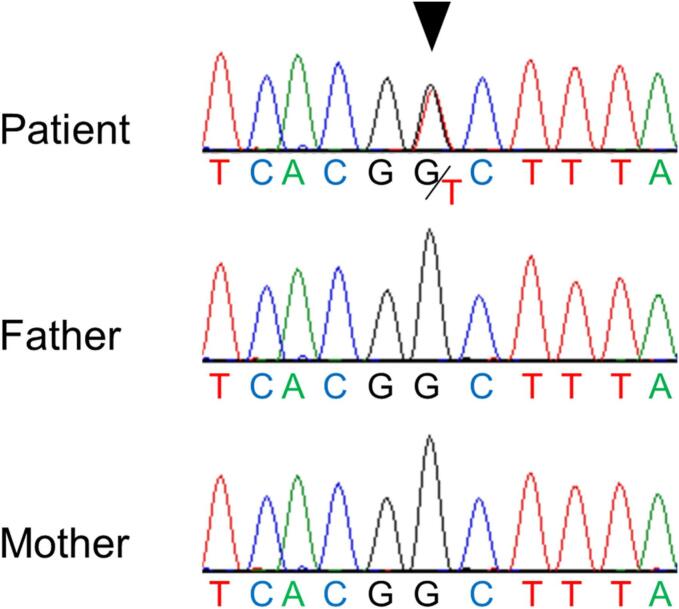
Results of Sanger electrophoresis of peripheral blood samples from the patient and her parents. Note the heterozygous mutation at chrX(GRCh37):g.19369472G > T, NM_001173454.1:c.479G > T, p.(Gly160Val), which was present in the patient but not in her parents (indicated by the arrowhead).

The urinary metabolome analysis showed that vitamin B1 supplementation reduced the total amount of urinary amino acids (i.e., glycine, alanine, serine, threonine, valine, leucine, isoleucine, lysine, arginine, histidine, methionine, cysteine, proline, glutamine, glutamic acid, asparagine, aspartic acid, tyrosine, phenylalanine, and tryptophan), and this trend was accelerated by the initiation of the ketogenic diet (Fig. 1B). The improvement in pan-aminoaciduria paralleled the decreases in urinary *N*-acetyl-β-D-glucosaminidase and β-2-microglobulin, which are known biomarkers of renal tubulopathy. However, most urinary amino acid concentrations including that of leucine, which is known to have an inhibitory effect on pyruvate oxidation, remained at least two-fold above the normal values for newborn female infants [19] before and even after the initiation of vitamin B1 supplementation and the ketogenic diet.

## 4 Discussion

In this study we performed serial metabolomic analyses of body fluids in an infant with PDHAD before and after the start of vitamin B1 supplementation and a ketogenic diet. Vitamin B1 improved the patient's renal tubular reabsorption, and the ketogenic diet resolved her central apnea and anemia. The ketogenic diet increased the total amounts of substrates in the TCA cycle, and the increases were in proportion to the ketogenic ratio. These findings suggested improved energetics in the renal, nervous, and hematopoietic systems. Taken together, the results obtained in this study provide molecular and clinical evidence that the combination of vitamin B1 supplementation and a ketogenic diet improves mitochondrial energetics in multiple organ systems.

The early institution of appropriate dietary and pharmacologic interventions is critical in treatable inborn errors of metabolism, and rapid and precise genetic diagnosis by whole genome sequencing is increasingly being utilized to make it possible to do so [20]. A recent study of genetic diagnosis in sick newborn infants reported that the neurometabolic phenotype provides the highest diagnostic yield of the various phenotypes [21]. As exemplified in this report, the neurological phenotype combined with metabolic abnormalities in sick newborn infants should prompt proactive rapid whole genome sequencing.

Vitamin B1 supplementation appeared to restore the renal mitochondrial energetics in the present patient. Indeed, the renal proximal tubules contain an abundancy of mitochondria second only to that in the central nervous system [22]. We speculated that vitamin B1 supplementation improved the mitochondrial energetics enough to resolve the renal tubular reabsorption in the present patient. Eventually, the addition of the ketogenic diet enabled the resolution of the apnea and anemia and reduced the lactate and pyruvate levels in the blood and CSF. These observations illustrate the utility of a urinary metabolome analysis for the systemic evaluation of the therapeutic efficacy of vitamin B1 supplementation and a ketogenic diet.

One potential biomarker for neuronal activity that we identified in the analyses in this study was glutamate. Glutamate is a major excitatory neurotransmitter in the central nervous system and functions as a metabolic hub between carbohydrate and amino acid metabolisms [23]. Decreased CSF glutamate levels have been reported in patients with neuropsychiatric disorders, including major depressive disorder [24,25]. Glutamate was absent in the CSF of our patient before treatment and was first detected accompanied by clinical improvement after the ketogenic ratio was increased to 3:1. It seems reasonable to speculate that glutamate in the CSF represents a biomarker for neuronal recovery.

In conclusion, vitamin B1 supplementation and a ketogenic diet improves mitochondrial energetics in multiple organ systems in PDHAD. The changes in carbohydrate and amino acids in body fluids, i.e., serum, CSF, and urine, in our patient were in proportion to the ketogenic ratio of the diet and paralleled the patient's clinical improvement. Rapid genetic diagnosis combined with therapeutic monitoring by means of metabolomic analyses is recommended in infants with inborn errors of carbohydrate metabolism, including PDHAD.

## Funding source

This research was supported by 10.13039/100009619Japan Agency for Medical Research and Development under Grant Number JP22gk0110038 (to TT), Japan Foundation for Pediatric Research, Grant No. 21-007 (to TT), Keio Gijuku Academic Development Funds (to TT), Kawano Masanori Memorial Public Interest Incorporated Foundation for Promotion of Pediatrics (to TT), Japan Society for the Promotion of Science under Grant-in-Aid for Challenging Exploratory Research, Grant Number JP22K19494, and Mother and Child Health Foundation (to TT). This research was also supported by World Premier Institute of BioQ2 for 10.13039/501100001697Keio University (to MS), and 10.13039/100009619Japan Agency for Medical Research and Development - Moonshot Project for Microbiome (to MS).

## Author contributions

Eri Ogawa drafted the first manuscript. Takako Hishiki, Noriyo Hayakawa, Hisato Suzuki, Kenjiro Kosaki, Makoto Suematsu reviewed and critically revised the manuscript. Toshiki Takenouchi conceived and organized the entire project and edited the final article.

## Declaration of Competing Interest

The authors have no conflicts of interest directly relevant to the content of this article.

## Data Availability

Data will be made available on request.
